# Tankyrase inhibition aggravates kidney injury in the absence of CD2AP

**DOI:** 10.1038/cddis.2016.217

**Published:** 2016-07-21

**Authors:** S Kuusela, H Wang, A A Wasik, H Suleiman, S Lehtonen

**Affiliations:** 1 Department of Pathology, University of Helsinki, Helsinki, Finland; 2 HHMI/Department of Pathology and Immunology, Washington University School of Medicine, St. Louis, MO, USA

## Abstract

Inappropriate activation of the Wnt/*β*-catenin pathway has been indicated in podocyte dysfunction and injury, and shown to contribute to the development and progression of nephropathy. Tankyrases, multifunctional poly(ADP-ribose) polymerase (PARP) superfamily members with features of both signaling and cytoskeletal proteins, antagonize Wnt/*β*-catenin signaling. We found that tankyrases interact with CD2-associated protein (CD2AP), a protein essential for kidney ultrafiltration as CD2AP-knockout (CD2AP−/−) mice die of kidney failure at the age of 6–7 weeks. We further observed that tankyrase-mediated total poly-(ADP-ribosyl)ation (PARylation), a post-translational modification implicated in kidney injury, was increased in mouse kidneys and cultured podocytes in the absence of CD2AP. The data revealed increased activity of *β*-catenin, and upregulation of lymphoid enhancer factor 1 (LEF1) (mediator of Wnt/*β*-catenin pathway) and fibronectin (downstream target of Wnt/*β*-catenin) in CD2AP−/− podocytes. Total PARylation and active *β*-catenin were reduced in CD2AP−/− podocytes by tankyrase inhibitor XAV939 treatment. However, instead of ameliorating podocyte injury, XAV939 further upregulated LEF1, failed to downregulate fibronectin and induced plasminogen activator inhibitor-1 (*PAI-1*) that associates with podocyte injury. In zebrafish, administration of XAV939 to CD2AP-depleted larvae aggravated kidney injury and increased mortality. Collectively, the data reveal sustained activation of the Wnt/*β*-catenin pathway in CD2AP−/− podocytes, contributing to podocyte injury. However, we observed that inhibition of the PARylation activity of tankyrases in the absence of CD2AP was deleterious to kidney function. This indicates that balance of the PARylation activity of tankyrases, maintained by CD2AP, is essential for normal kidney function. Furthermore, the data reveal that careful contemplation is required when targeting Wnt/*β*-catenin pathway to treat proteinuric kidney diseases associated with impaired CD2AP.

The glomerular filtration barrier consists of fenestrated endothelial cells, glomerular basement membrane and glomerular epithelial cells or podocytes. Disruption of the filtration barrier leads to proteinuria, which may progress to renal failure. Podocytes are terminally differentiated cells with a large cell body and interdigitating foot processes, which rest on the glomerular basement membrane. Podocyte injury has been shown to have an important role in the development of proteinuria as the loss of function of several podocyte proteins has been shown to lead to podocyte foot process effacement and impaired glomerular ultrafiltration.^1^

Adapter protein CD2-associated protein (CD2AP) is essential for the maintenance of glomerular filtration.^2^ CD2AP was originally characterized as an interaction partner of T-cell adhesion molecule CD2,^3^ and as a protein upregulated during mesenchyme to epithelium differentiation in the developing kidney.^4^ In the glomerulus, CD2AP interacts with nephrin,^5^ the key component of the slit diaphragm interconnecting the neighboring podocyte foot processes.^6^ CD2AP regulates cytoskeletal remodeling,^7^ apoptosis^8^ and vesicular trafficking.^9^

Tankyrases 1 and 2 are members of the poly(ADP-ribose) (PAR) polymerase family.^10, 11^ They catalyze poly(ADP-ribosyl)ation (PARylation), a post-translational modification targeting tankyrases themselves or their interaction partners, subject the target proteins to ubiquitination and proteasomal degradation,^10^ and thereby modulate diverse cellular processes.^12^ During recent years, several new interaction partners and functions have been characterized for tankyrases;^13, 14, 15^ however, the possible involvement of tankyrases in regulating podocyte function has remained unknown. PARylation activity of tankyrases is involved in regulating the activity of the canonical Wnt/*β*-catenin signaling pathway.^16^ When the pathway is inactive, *β*-catenin is phosphorylated and targeted to degradation by the *β*-catenin destruction complex. Tankyrases PARylate AXIN in the destruction complex, stimulate its degradation, and thus enable dephosphorylation of *β*-catenin and its consequent translocation to the nucleus where it together with T-cell factor/lymphoid enhancer factor (TCF/LEF) family of co-activators drives Wnt-specific transcriptional programs.^17^ In the adult kidney, Wnt/*β*-catenin pathway has been considered to be silent. Inappropriate activation of the pathway has been indicated in podocyte dysfunction and injury,^18^ and in many kidney diseases such as diabetic nephropathy and adriamycin-induced nephropathy.^19, 20, 21, 22^

Here, we show that tankyrases are novel interaction partners of CD2AP, and that CD2AP is a negative regulator of tankyrases. We demonstrate that activation of *β*-catenin accompanied by elevation of total PARylation associates with podocyte injury in the absence of CD2AP. However, inhibition of tankyrases in the absence of CD2AP enhances the expression of podocyte injury markers and aggravates kidney damage.

## Results

### Tankyrases 1 and 2 form a complex with CD2AP

**Figure 1:**
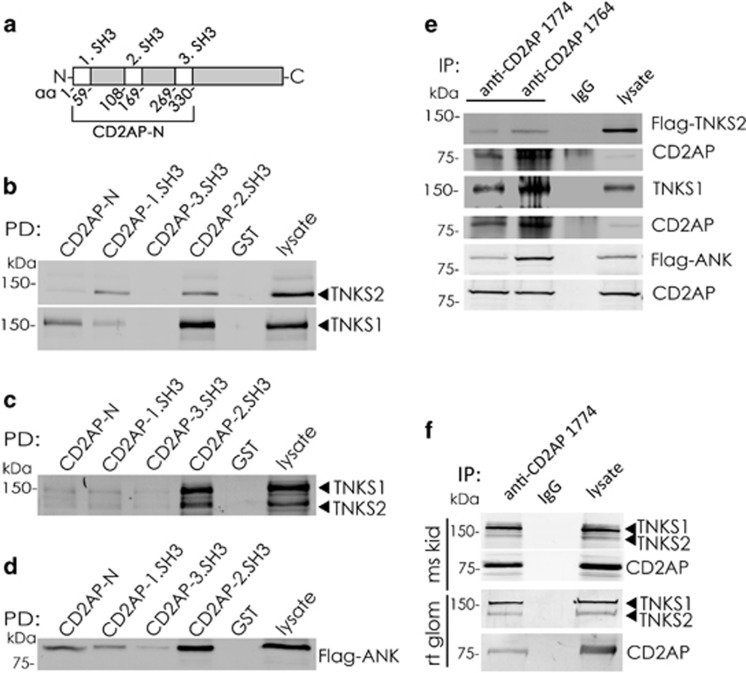
Tankyrases form a complex with CD2AP. (**a**) Schematic cartoon illustrating the amino acids covered by the SH3 domains and the N-terminus of CD2AP. (**b**) Tankyrase 1 (TNKS1) and tankyrase 2 (TNKS2) transiently overexpressed in Hek293 cells are pulled down with GST-CD2AP, but not with GST alone. The strongest interaction is observed between tankyrases and the GST-2.SH3 domain of CD2AP. (**c**) Tankyrase 1 and tankyrase 2 endogenously expressed in rat glomeruli are pulled down with GST-2.SH3 domain of CD2AP, but not with GST alone. (**d**) The Flag-tagged ankyrin domains (Flag-ANK) of tankyrase 2 transiently overexpressed in Hek293 cells are pulled down with GST-2.SH3 domain of CD2AP, but not with GST alone. (**e**) Flag-tagged tankyrase 2, tankyrase 1 and Flag-tagged ankyrin domains of tankyrase 2 co-immunoprecipitate with CD2AP 1774 and 1764 antibodies, but not with rabbit IgG in Hek293 cells transiently transfected with Flag-tankyrase 2, tankyrase 1 or Flag-tagged ankyrin domains of tankyrase 2. (**f**) Tankyrase 1 and tankyrase 2 endogenously expressed in mouse kidneys (ms kid) and rat glomeruli (rt glom) co-immunoprecipitate with CD2AP 1774 antibody, but not with rabbit IgG. Hek293 or glomerular lysates (50 *μ*g) are included as a control. The experiments were repeated three times with similar results

We performed a yeast two-hybrid screening on a rat glomerular library to identify proteins that interact with the NH_2_ terminus of CD2AP containing three SH3 domains. The screening identified a clone that covered amino acids 1–190 of tankyrase 2. Pull-down assays with the NH_2_ terminus or individual SH3 domains of CD2AP fused with GST (Figure 1a, Supplementary Figure S1a) confirmed the interaction (Figure 1b). Immunoblotting revealed that the 2.SH3 domain of CD2AP pulled down tankyrase 2 and tankyrase 1 overexpressed in HEK293T cells (Figure 1b,Supplementary Figure S1b). Also endogenous tankyrases were pulled down with the 2.SH3 domain of CD2AP from rat glomerular lysate (Figure 1c). The interaction between CD2AP and tankyrase 2 was mediated by the ankyrin domains (Figure 1d). Both full-length tankyrases and the ankyrin domains of tankyrase 2 were co-immunoprecipitated with endogenous CD2AP (Figure 1e). Furthermore, CD2AP co-immunoprecipitated endogenous tankyrases from mouse kidney and isolated rat glomerular lysates (Figure 1f), confirming that CD2AP and tankyrases form a physiological complex.

### Tankyrases 1 and 2 are expressed in podocytes

**Figure 2:**
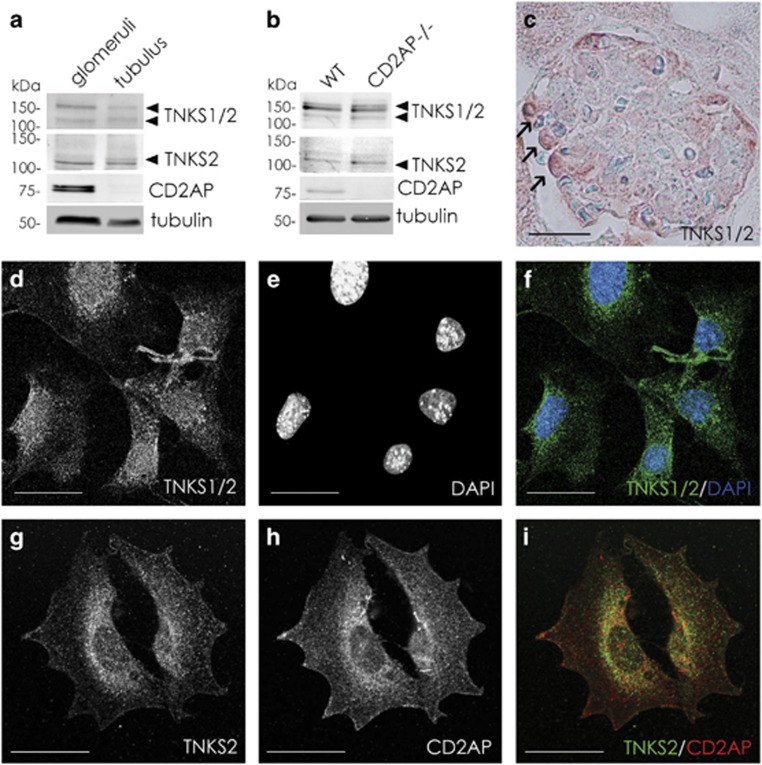
Tankyrases are expressed in podocytes. (**a**) Immunoblotting of rat glomerular and tubular fractions with antibodies that recognize tankyrases 1 and 2 (TNKS1/2) or tankyrase 2 (TNKS2) indicates that tankyrase 1 and tankyrase 2 are expressed in both glomerular and tubular fractions. CD2AP concentrates in the glomeruli. Tubulin is included as a control. (**b**) Immunoblotting indicates that tankyrases 1 and 2 are expressed in wild-type (WT) and CD2AP−/− podocyte lysates. CD2AP is detected only in the WT podocytes as expected. Tubulin is included as a control. (**c**) Immunoperoxidase staining of mouse kidney section with an antibody that recognizes both tankyrases 1 and 2 (TNKS1/2) reveals that tankyrases are expressed in glomeruli, where they localize in podocytes (arrows). (**d**–**f**) Immunofluorescence staining of WT mouse podocytes shows that tankyrases primarily localize to the perinuclear region and nucleus. DAPI labels the nuclei. (**g**–**i**) Double immunofluorescence staining shows that tankyrase 2 (**g**) and CD2AP (**h**) partially colocalize in the perinuclear region in WT mouse podocytes (**i**). Scale bar: (**c**) 50 *μ*m; (**d**–**i**) 20 *μ*m

Tankyrases are expressed in both glomeruli and tubuli, whereas CD2AP is found predominantly in the glomeruli (Figure 2a). Tankyrases are also expressed in cultured wild-type (WT) and CD2AP-knockout (CD2AP−/−) podocytes (Figure 2b). In kidney tankyrases localize in podocytes (Figure 2c), and in cultured podocytes tankyrases are distributed both in the nucleus and in the cytoplasm (Figures 2d–f) where they partially colocalize with CD2AP (Figures 2g–i).

### Tankyrase-mediated total PARylation is increased in CD2AP−/− podocytes *in vitro*

**Figure 3:**
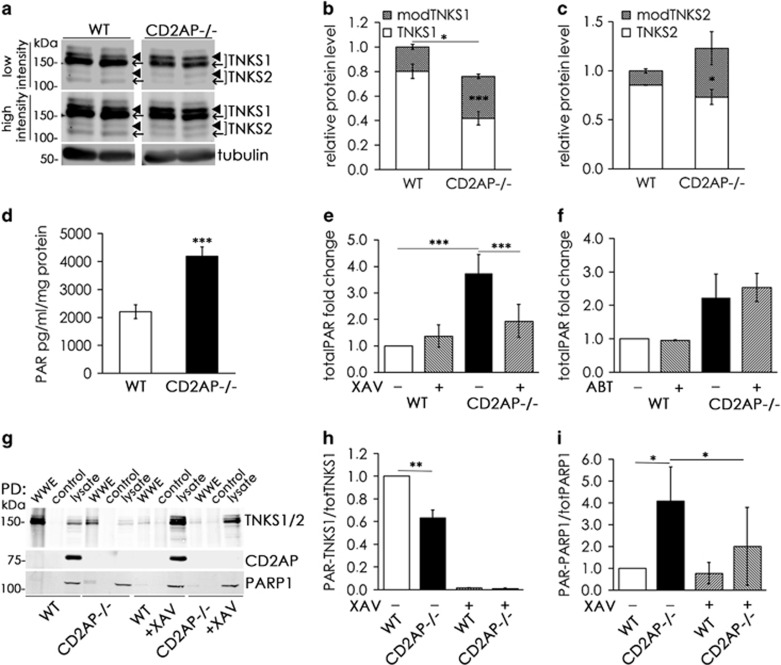
Tankyrase-mediated total PARylation is increased in CD2AP−/− podocytes *in vitro*. (**a**) Wild-type (WT) and CD2AP−/− podocyte cell lysates were immunoblotted with an antibody that recognizes both tankyrase 1 (TNKS1) and tankyrase 2 (TNKS2). The expected molecular weights of tankyrase 1 and tankyrase 2 are 150 and 130 kDa, respectively. Two replicates of WT and CD2AP−/− podocyte lysates are shown. Arrowheads indicate slower migrating, modified forms and arrows indicate the non-modified forms of tankyrases 1 and 2. The high intensity image is shown to better visualize tankyrase 2, which is expressed at a lower level than tankyrase 1 in podocytes. Tubulin is included as a loading control. (**b**) Quantification of three replicate blots as in (**a**). The bars (white and gray parts together) indicate the total level of tankyrase 1, and the gray part of the bar indicates the fraction of tankyrase 1 that is post-translationally modified (the upper band). Quantification indicates that tankyrase 1 is downregulated in CD2AP−/− podocytes, but its post-translational modification is increased. (**c**) Quantification of three replicate blots as in (**a**) indicates that tankyrase 2 is expressed at a similar level in WT and CD2AP−/− podocytes, but its post-translational modification is increased in the absence of CD2AP. (**d**) Total PARylation activity is increased in cultured CD2AP−/− podocytes compared with WT podocytes. (**e**) Inhibition of tankyrases with a tankyrase-specific small molecule inhibitor XAV939 for 18 h (3 *μ*M) lowers the total PARylation in cultured CD2AP−/− podocytes. (**f**) Treatment of WT and CD2AP−/− podocytes with PARP1/2-specific inhibitor ABT-888 for 18 h (3*μ*M) does not affect total PARylation in either cell line. (**g**) Pull-down assay from WT and CD2AP−/− podocytes with PAR-polymer-binding-resin (WWE affinity resin) followed by western blotting with antibodies recognizing tankyrases 1 and 2, PARP1 and CD2AP indicates that tankyrase 1 is PARylated in podocytes. PARP1 is weakly PARylated, whereas CD2AP is not PARylated. Control pull down (control) was performed with WWE domain harboring a mutation, which renders the domain unable to bind PAR. Cell lysates (30 *μ*g) were included as controls. (**h** and **i**) Quantification of three replicate blots as in (**g**) reveals that PARylation of tankyrase 1 is reduced (**h**) and PARylation of PARP1 is increased (**i**) in CD2AP−/− podocytes. The amounts of tankyrase 1 and PARP1 in the pull downs with WWE domain were normalized to the amounts of tankyrase 1 and PARP1 in the lysates, respectively. Bars show the mean and error bars the ±standard error of mean (S.E.M.) of three independent experiments with three biological replicates. *P-*values were calculated with one-way ANOVA (*P<0.05; **P<0.01; ***P<0.001)

Immunoblotting of lysates of WT and CD2AP−/− podocytes revealed multiple bands for both tankyrase 1 and tankyrase 2 (Figure 3a). Tankyrase 1 is expected to migrate at 150 kDa and tankyrase 2 at 130 kDa. Quantification indicated that the total level of tankyrase 1 is decreased but its slower migrating, modified form is increased in CD2AP−/− podocytes compared with WT podocytes (Figure 3b). The total level of tankyrase 2 was similar between WT and CD2AP−/− podocytes; however, the modified form of tankyrase 2 was increased in the absence of CD2AP (Figure 3c).

Tankyrase 1 is modified by phosphorylation,^23^ and phosphatase (CIP) treatment of WT and CD2AP−/− cell lysates confirmed that the slower migrating form of tankyrase 1 is phosphorylated in podocytes (Supplementary Figure S2). Tankyrase 1 phosphorylation can enhance its PARylation activity^23^ or autoPARylation.^16^ We therefore examined whether total PARylation is increased in CD2AP−/− podocytes. Indeed, the absence of CD2AP increased total PARylation in podocytes compared with WT cells (Figure 3d). Treatment of CD2AP−/− podocytes with a tankyrase-specific inhibitor XAV939 reduced total PARylation (Figure 3e) whereas ABT-888 (veliparib), a PARP1 and PARP2 (poly(ADP-ribose) polymerase 1 and 2)-specific inhibitor,^24, 25^ had no effect (Figure 3f). The effectiveness of ABT-888 was confirmed by its ability to inhibit H_2_O_2_-induced PARP1-mediated PARylation (Supplementary Figure S3). These data confirm that the increase in total PARylation in the absence of CD2AP is due to the increased activity of tankyrases.

Tankyrases typically PARylate their interaction partners.^10, 13, 14, 16, 23, 26^ Pull down with PAR-affinity resin from WT podocyte lysates showed that CD2AP is not PARylated (Figure 3g). Interestingly, we detected less PARylated tankyrases in CD2AP−/− podocytes compared with WT podocytes (Figures 3g and h). One of the known PARylation targets of tankyrases is PARP1.^27^ PARP1 is an enzyme involved in cell differentiation, survival and transformation, and its main function is to repair single-stranded DNA breaks.^28^ Lack of CD2AP increased the PARylation of PARP1 in podocytes and tankyrase inhibitor XAV939 treatment reduced it (Figures 3g and i), indicating that PARylation of PARP1 in the absence of CD2AP is tankyrase mediated. Additionally, inhibitor treatment reduced PARylation of tankyrases in WT and CD2AP−/− podocytes confirming the effectiveness of the inhibitor (Figures 3g and h). Collectively, the absence of CD2AP increases tankyrase-mediated PARylation, and one of the PARylation targets of tankyrases is PARP1.

### Total PARylation is increased in CD2AP−/− podocytes *in vivo*

**Figure 4:**
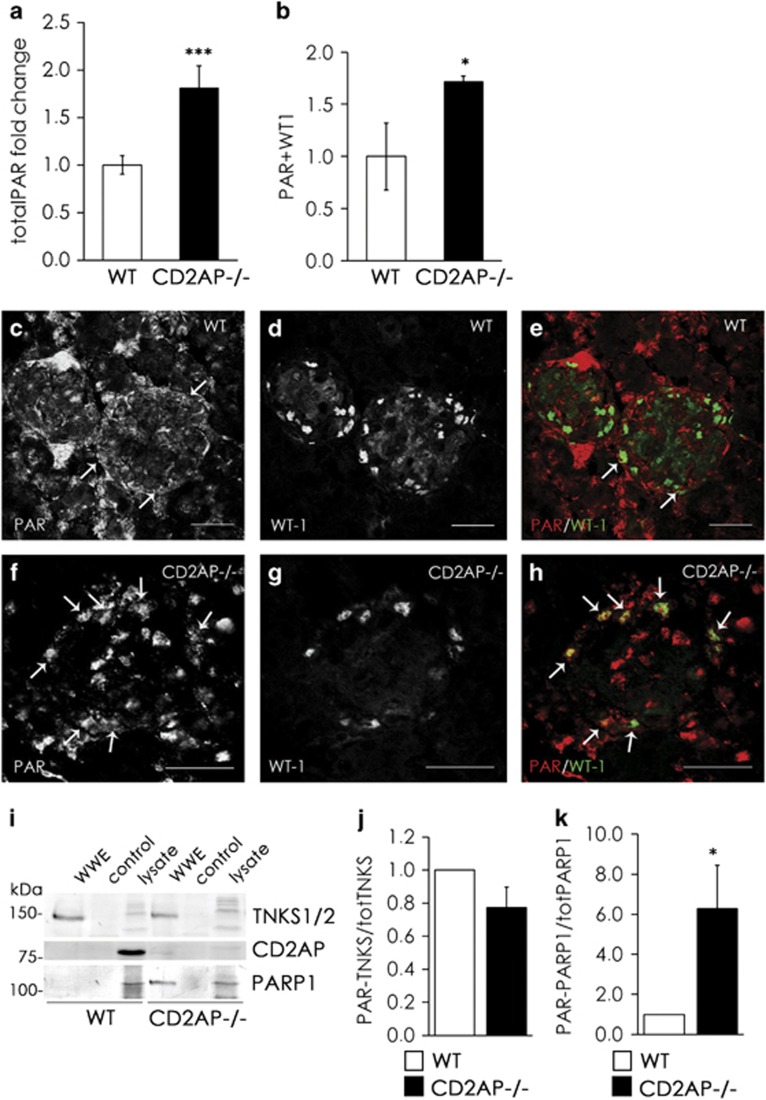
Total PARylation is increased in CD2AP−/− podocytes *in vivo*. (**a**) Total PARylation is increased in the kidneys of CD2AP−/− mice compared with WT mice. PARylation was measured from kidney lysates of 7 WT and 11 CD2AP−/− mice. (**b**) The number of PAR-positive podocytes is significantly higher in CD2AP−/− kidneys compared with WT kidneys. Kidney sections of three wild-type and three CD2AP−/− mice were double labeled with anti-PAR and anti-WT1 IgGs. Numbers of PAR-positive podocytes were calculated from 20 glomeruli/animal. (**c**–**h**) Representative images of WT (**c**–**e**) and CD2AP−/− (**f**–**h**) kidney sections labeled with anti-PAR (**c** and **f**) and anti-WT1 (**d** and **g**) IgGs. Merged images (**e** and **h**) show an increase in PAR-positive podocytes (arrows) in the absence of CD2AP (**h**). (**i**) Pull-down assay from WT and CD2AP−/− kidney lysates with PAR-polymer-binding-resin (WWE) followed by western blotting with antibodies recognizing tankyrases 1 and 2, PARP1 and CD2AP indicates that tankyrase 1 is PARylated in mouse kidneys. PARP1 shows negligible level of PARylation in WT kidneys, but its PARylation increases in the absence of CD2AP. CD2AP is not PARylated *in vivo*. (**j** and **k**) Quantification of three replicate blots as in (**i**) showed that there is no difference in the PARylation of tankyrase 1 (**j**) and that there is more PARylated PARP1 (**k**) in CD2AP−/− kidneys when compared with WT. The amount of tankyrase 1 and PARP1 in the pull down with WWE domain was normalized to the amount of tankyrase 1 and PARP1, respectively, in the lysate. Bars show the mean and error bars the ±S.E.M. of three independent experiments. *P*-values were calculated with one-way ANOVA (*P<0.05; ***P<0.001). Scale bar: (**c**–**h**) 25 *μ*m

Shih *et al.*^2^ showed that CD2AP-deficient mice develop high-grade albuminuria and exhibit lesions typical of focal segmental glomerulosclerosis (FSGS) by 3–4 weeks of age. Kidney failure results in the death of the animals by the age of 6–7 weeks.^2, 8^ We examined whether we observe an increase in total PARylation, shown to associate with kidney injury in diabetes,^29^ in the kidneys of CD2AP-deficient mice at 3 weeks of age. Indeed, we observed that CD2AP deficiency leads to increased total PARylation in the kidneys compared with WT kidneys (Figure 4a). Double labeling of CD2AP−/− and WT kidneys with anti-PAR and anti-WT1 antibodies revealed that the number of podocytes with PAR-positive staining was significantly higher in the absence of CD2AP (Figure 4b), and the staining for PAR was prominent in the nuclei (Figures 4c–h,Supplementary Figure S4). CD2AP was not PARylated in the kidney (Figure 4i). PARylation of tankyrases was similar in WT and CD2AP−/− kidneys (Figures 4i and j), whereas the absence of CD2AP increased PARP1 PARylation (Figures 4i and k). Taken together, these data are in line with the observations in cultured podocytes, showing that the absence of CD2AP increases PARylation in podocytes also *in vivo*.

### Absence of CD2AP leads to the accumulation of active *β*-catenin, *Lef1* and *TCF -1* in podocytes

**Figure 5:**
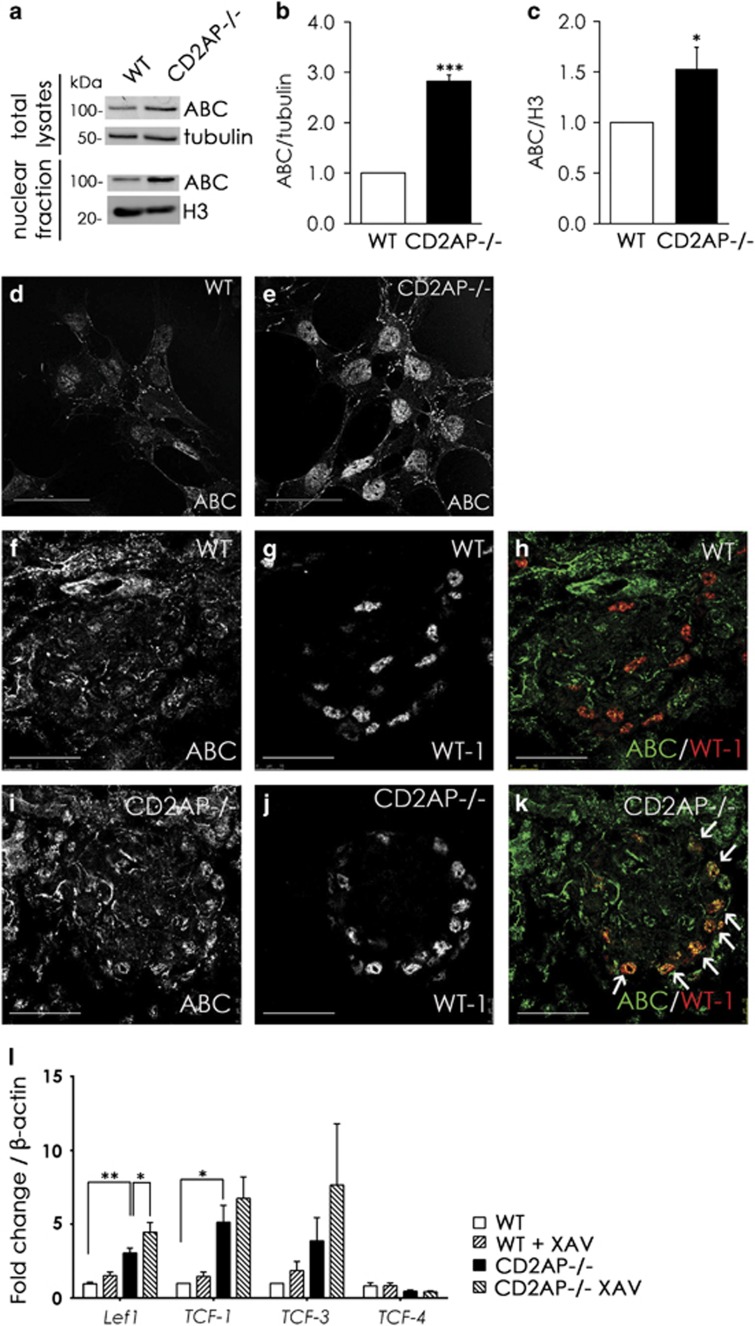
Active *β*-catenin and its co-factors *Lef1* and *TCF-1* are upregulated in the absence of CD2AP. (**a**) Immunoblotting of wild-type (WT) and CD2AP−/− podocytes for active *β*-catenin (ABC) in total lysates and nuclear fractions shows upregulation of active *β*-catenin. Tubulin and histone H3 (H3) are included as loading controls. (**b** and **c**) Quantification of three replicate blots as in (**a**) indicates accumulation of active *β*-catenin in CD2AP−/− podocytes (**b**) and in the nuclei of CD2AP−/− podocytes (**c**). (**d** and **e**) Immunofluorescence staining indicates that active *β*-catenin concentrates in the nucleus of CD2AP−/− podocytes. (**f**–**k**) Representative images of WT (**f**–**h**) and CD2AP−/− (**i**–**k**) kidney sections labeled with active *β*-catenin (ABC) (**f** and **i**) and WT1 (**g** and **j**) IgGs. Merged images (**h** and **k**) show an increase in active *β*-catenin-positive podocytes (arrows) in the absence of CD2AP (**k**). (**l**) Quantitative RT-PCR analysis indicates that *Lef1* and *TCF-1* are upregulated in CD2AP−/− podocytes compared with WT podocytes. There is no difference in *TCF-3* and *TCF-4* expression. Tankyrase inhibitor XAV939 treatment of CD2AP−/− podocytes further upregulates *Lef1* and *TCF-1*. Bars show the mean and error bars the ±S.E.M. of three independent experiments. *P*-values were calculated with one-way ANOVA (*P<0.05; **P<0.01; ***P<0.001). Scale bar: (**d**–**k**) 25 *μ*m

Owing to the observed overactivity of tankyrases, we hypothesized that one of the mechanisms contributing to podocyte injury in the absence of CD2AP is the activation of Wnt/*β*-catenin pathway. Indeed, active *β*-catenin was upregulated in CD2AP−/− podocytes compared with WT podocytes without exogenous Wnt stimulation (Figure 5a), and accumulated in the nucleus (Figures 5a–e). This was also observed in the glomerular podocytes of CD2AP−/− mouse kidneys when compared with WT (Figures 5f–k).

TCF/LEF family of transcription factors are the major mediators of Wnt signaling and their expression is upregulated by stabilized *β*-catenin.^30^ qRT-PCR indicated that the absence of CD2AP leads to upregulation of *Lef1* and *TCF-1* mRNAs in podocytes (Figure 5l), whereas *TCF-3* and *TCF-4* had similar expression. Interestingly, treatment with tankyrase inhibitor, Wnt/*β*-catenin pathway antagonist XAV939, had no effect on *TCF-1, TCF-3* or *TCF-4* expression; however, it significantly increased *Lef1* expression in CD2AP−/− podocytes (Figure 5l).

**Figure 6:**
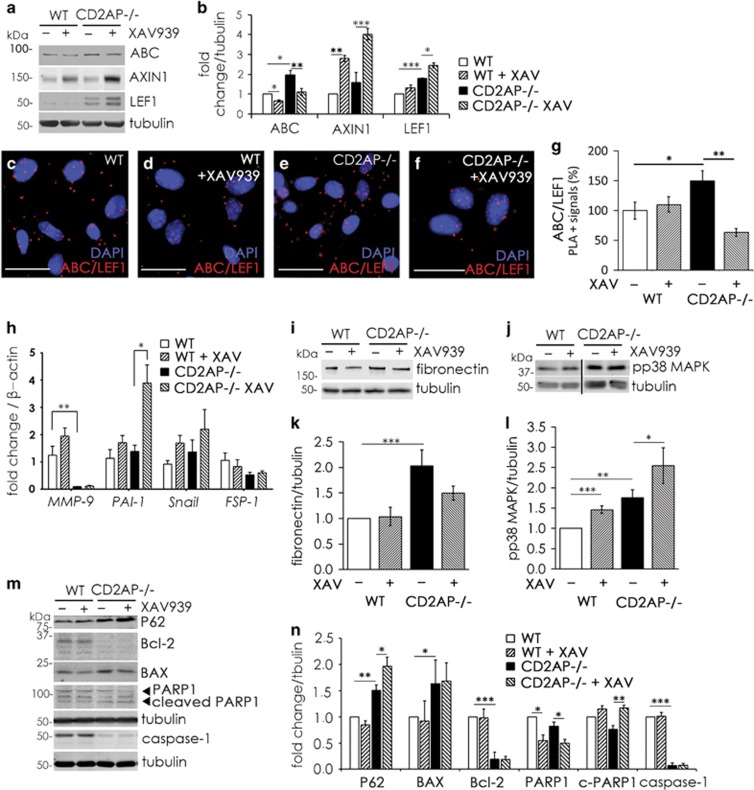
Tankyrase inhibitor treatment upregulates LEF1, fails to downregulate fibronectin and increases pro-apoptotic signaling in the absence of CD2AP. (**a**) Immunoblotting of wild-type (WT) and CD2AP−/− podocyte lysates for active *β*-catenin (ABC), AXIN1 and LEF1 with or without tankyrase inhibitor XAV939 treatment. Tubulin is included as a loading control. (**b**) Quantification of three replicate blots as in (**a**) indicates that tankyrase inhibitor treatment reduces active *β*-catenin and upregulates AXIN1 in WT and CD2AP−/− podocytes. LEF1 is upregulated in CD2AP−/− podocytes and is further upregulated with tankyrase inhibitor treatment. (**c**–**f**) Representative images of *in situ* proximity ligation assay (PLA) with ABC and LEF1 antibodies without (**c** and **d**) and with (**e** and **f**) tankyrase inhibitor XAV939 treatment. The red fluorescent dots indicate interaction between ABC and LEF1. DAPI is used as a nuclear counterstain. (**g**) Quantification of three independent PLA experiments shows that there is more interaction of active *β*-catenin and LEF1 in the CD2AP−/− podocytes compared with WT podocytes, and that this interaction is downregulated with tankyrase inhibitor treatment. (**h**) Quantitative RT-PCR analysis indicates that *MMP-9* is downregulated in CD2AP−/− podocytes compared with WT podocytes. The expression of *PAI-1, Snail1* and *FSP-1* does not differ between the cell lines. XAV939 treatment of CD2AP−/− podocytes upregulates *PAI-1*, and has no significant effect on *MMP-9*, *Snail1* and *FSP-1* in CD2AP−/− podocytes. (**i**) Immunoblotting for fibronectin in WT and CD2AP−/− podocytes treated or not with tankyrase inhibitor XAV929. (**j**) Immunoblotting with an antibody against phosphorylated p38 MAPK (pp38 MAPK) in WT and CD2AP−/− podocytes treated or not with tankyrase inhibitor XAV939. (**k**) Quantification of three independent blots as in (**i**) shows that fibronectin is upregulated in CD2AP−/− podocytes, and that tankyrase inhibitor treatment does not significantly downregulate its expression in CD2AP−/− podocytes. (**l**) Quantification of three replicate blots as in (**j**) shows that phosphorylation of p38 MAPK is increased in the absence of CD2AP. Tankyrase inhibitor further upregulates the phosphorylation of p38 MAPK in the absence of CD2AP, and also upregulates phosphorylation of p38 MAPK in WT podocytes. (**m**) Immunoblotting for P62, BAX, Bcl-2, PARP1 and caspase-1 in WT and CD2AP−/− podocytes. Tubulin was used as a loading control. (**n**) Quantification of three replicate blots as in (**m**) shows the effects of XAV939 treatment on the indicated proteins. Bars show the mean and error bars the ±S.E.M. of three independent experiments with three biological replicates. *P*-values were calculated with one-way ANOVA (*P<0.05; **P<0.01; ***P<0.001). Scale bar: (**c**–**f**) 25 *μ*m

XAV939 inhibits tankyrases and stabilizes AXIN1, which enhances degradation of active *β*-catenin.^17^ As expected, XAV939 downregulated active *β*-catenin and increased the levels of AXIN1 in both WT and CD2AP−/− podocytes (Figures 6a and b). LEF1 was upregulated in CD2AP−/− podocytes also at the protein level, and the upregulation was further enhanced by tankyrase inhibition (Figures 6a and b). Immunofluorescence staining confirmed that XAV939 treatment downregulates active *β*-catenin in both WT and CD2AP−/− podocytes and upregulates LEF1 in CD2AP−/− podocytes (Supplementary Figure S5). Active *β*-catenin binds to LEF1 to form transcription complexes to activate target gene expression.^31^ In line with this, proximity ligation assay (PLA) revealed that there is more interaction between LEF1 and active *β*-catenin in CD2AP−/− podocytes compared with WT podocytes (Figures 6c, d and g). XAV939 treatment decreased the prevalence of the interaction in CD2AP−/− podocytes, possibly due to downregulation of active *β*-catenin (Figures 6e–g). Taken together, the absence of CD2AP leads to sustained activation of *β*-catenin in podocytes, which upregulates LEF1. Interestingly, XAV939 treatment further upregulated LEF1 in CD2AP−/− podocytes.

### Tankyrase inhibition in CD2AP−/− podocytes aggravates pro-apoptotic signaling and podocyte injury

Several Wnt-signaling target genes, including *Snail1*, plasminogen activator inhibitor-1 (*PAI-1*), fibroblast-specific protein 1 (*FSP-1*), matrix metalloproteinase 9 (*MMP-9*) and fibronectin, have been shown to be upregulated in kidney injury.^18, 20^ However, we observed no significant difference in the expression levels of *Snail1*, *PAI-1* and *FSP-1* between CD2AP−/− and WT podocytes (Figure 6h). Interestingly, the absence of CD2AP significantly decreased the expression of *MMP-9* (Figure 6h), and increased the expression of fibronectin (Figures 6i and k). The data thus reveal regulation of a specific set of Wnt-target genes in the absence of CD2AP.

XAV939 treatment had no effect on *MMP-9*, *Snail1 or FSP-1* expression in WT or CD2AP−/− podocytes (Figure 6h), and fibronectin expression remained elevated in CD2AP−/− podocytes (Figures 6i and k). Furthermore, XAV939 significantly upregulated *PAI-1* in CD2AP−/− podocytes (Figure 6h). As also previously shown,^8^ the absence of CD2AP increased the expression of apoptotic p38 MAPK phosphorylation (Figures 6j and l). XAV939 treatment further increased p38 MAPK phosphorylation (Figures 6j and l). Also insulin-stimulated phosphorylation of anti-apoptotic AKT was reduced by XAV939 treatment in podocytes lacking CD2AP (Supplementary Figure S7). Additionally, we investigated the expression of apoptotic markers in CD2AP−/− podocytes with and without tankyrase inhibition. The absence of CD2AP increased the expression of pro-apoptotic Bcl-2-associated X protein (BAX) (Figures 6m and n), whereas the anti-apoptotic B-cell lymphoma 2 protein (Bcl-2), a dual regulator of apoptosis and autophagy, was downregulated. The autophagy signaling adaptor protein p62 (sequestosome-1), which in a context-dependent manner, has a role in the decision of the cells undergoing autophagy to either survive or die, was upregulated in the absence of CD2AP and XAV939 further increased its expression (Figures 6m and n). Full-length caspase-1 was downregulated in the absence of CD2AP (Figures 6m and n). XAV939 treatment downregulated full-length PARP1 in both WT and CD2AP−/− podocytes, and increased the expression of cleaved, apoptotic form of PARP1 in the absence of CD2AP (Figures 6m and n). The data indicate that inhibition of tankyrases in the absence of CD2AP aggravates podocyte injury by increasing pro-apoptotic signaling and upregulating LEF1 and *PAI-1* (see cartoon in Figure 8).

### Inhibition of tankyrases in *cd2ap* knockdown zebrafish aggravates kidney injury

**Figure 7:**
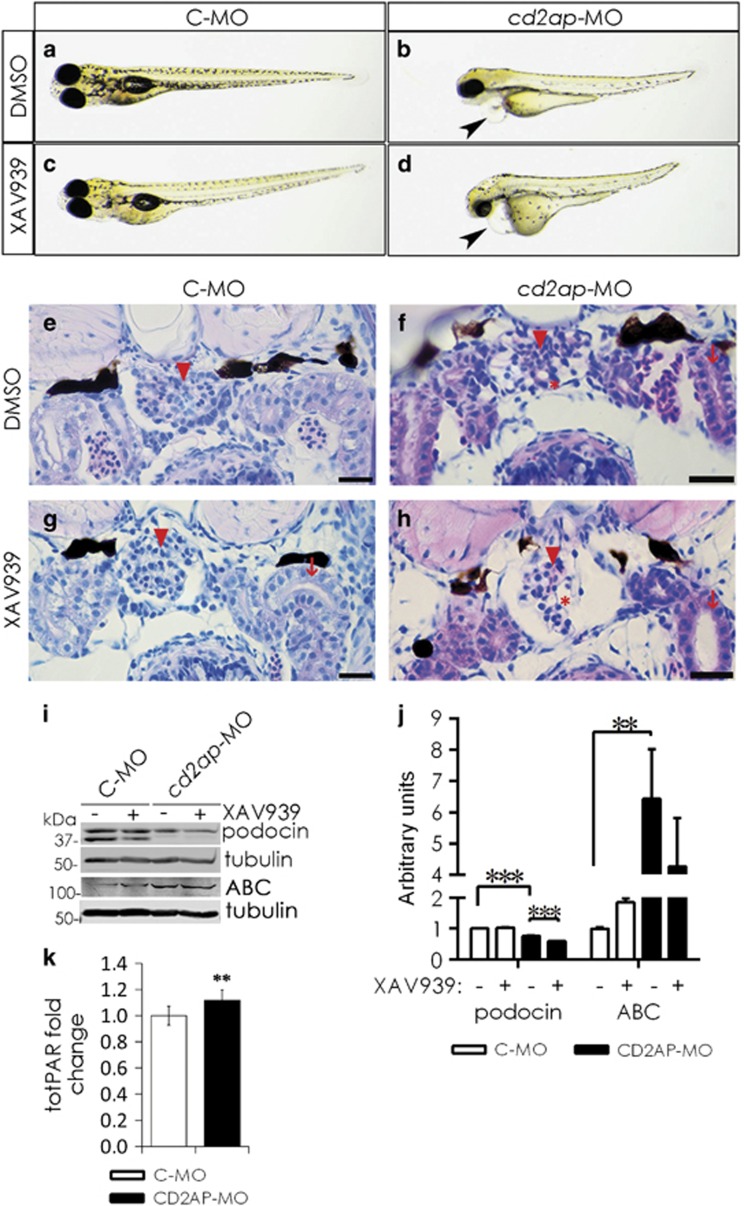
Simultaneous disruption of *cd2ap* expression and tankyrase activity aggravates kidney injury in zebrafish larvae. (**a** and **b**) Knockdown of *cd2ap* with morpholino antisense oligonucleotides (*cd2ap*-MO) in zebrafish causes pericardial edema (arrowhead) at 5 days post fertilization (dpf) indicative of kidney dysfunction (**b**). Control-MO (C-MO)-injected larvae appear developmentally normal (**a**). (**c** and **d**) Inhibition of tankyrase activity with XAV939 in the *cd2ap* morphant between 3 and 5 dpf induces yolk sac edema and more severe pericardial edema (**d**, arrowhead) than knockdown of *cd2ap* alone (**b**), whereas XAV939 treatment has no effect on the control morphant (**c**). (**e** and **g**) Histological sections of 5 dpf control morphant (**e**) and control morphant treated with XAV939 (**g**) show normal glomeruli (arrowheads) and proximal convoluted tubules (arrows). (**f**) *cd2ap* morphant shows a disturbed glomerular structure (arrowhead) with cysts (asterisk). (**h**) Inhibition of tankyrase activity with XAV939 in *cd2ap* morphants leads to even more destructed glomerular structure with a dilated (asterisk) and stretched septal glomerulus in the midline (arrowhead) and dilated proximal convoluted tubule (arrow). (**i**) Immunoblotting for podocin and active *β*-catenin (ABC) in control morphants (C-MO) and *cd2ap* morphants (*cd2ap*-MO) with and without XAV939 treatment. *α*-Tubulin is included as a control. (**j**) Quantification of three replicate blots as in (**i**) shows significant downregulation of podocin and upregulation of ABC in *cd2ap* morphants compared with control morphants. XAV939 treatment further downregulates podocin in *cd2ap* morphants. (**k**) Knockdown of *cd2ap* with *cd2ap*-MO in zebrafish leads to an increase in total PARylation at 5 dpf compared with control-MO treated zebrafish. *P*-values were calculated with one-way ANOVA (**P<0.01; ***P<0.001). Scale bar: (**e**–**h**) 25 *μ*m

To analyze whether inhibition of tankyrases increases podocyte injury in the absence of CD2AP *in vivo*, we knocked down *cd2ap* with morpholino antisense oligonucleotides (MOs) in zebrafish, previously shown to result in pronephric kidney dysfunction,^32^ coupled with tankyrase inhibition. At 5 days post fertilization (dpf), 60–68% of *cd2ap*-MO- and *cd2ap*-MO and tankyrase inhibitor-treated larvae exhibited pericardial edema indicative of kidney dysfunction (Figures 7a and b,Supplementary Figure S6). Notably, simultaneous suppression of *cd2ap* expression and tankyrase activity increased the number of larvae displaying both pericardial and yolk sac edema from 13 to 27% (Figure 7d,Supplementary Figure S6) and increased mortality from 3 to 17% compared with suppression of *cd2ap* expression alone (Figures 7b and d,Supplementary Figure S6). Downregulation of *cd2ap* alone or XAV939 treatment of control-MO-injected larvae had no effect on viability (Supplementary Figure S6); and XAV939 treatment of control-MO-injected larvae caused no identifiable malformation (Figure 7c).

Control-MO-injected larvae without or with XAV939 treatment showed well-developed glomeruli and pronephric ducts (Figures 7e and g). The glomeruli of *cd2ap*-MO-treated larvae exhibited disturbed glomerular structures with distended capillary loops (Figure 7f), whereas the *cd2ap* knockdown larvae treated with XAV939 showed even more disturbed glomerular structures with dilated Bowman's space and proximal convoluted tubules and stretched, septal glomeruli at the midline (Figure 7h) indicating severe kidney damage. Suppression of *cd2ap* downregulated podocin (Figures 7i and j), a protein essential for glomerular structure and function.^33^ Notably, XAV939 treatment further downregulated podocin in *cd2ap*-MO-injected larvae, but had no effect in control-MO-injected larvae (Figures 7i and j). In line with the *in vitro* experiments, active *β*-catenin was upregulated in *cd2ap*-MO-injected larvae (Figures 7i and j). Additionally, suppression of *cd2ap* increased total PAR levels compared with controls (Figure 7k). Collectively, the data indicate that suppression of *cd2ap* coupled with inhibition of tankyrases leads to more severe kidney damage than knockdown of *cd2ap* alone and increases mortality of the larvae.

## Discussion

Canonical Wnt/*β*-catenin signaling mediates podocyte injury and the development of proteinuria,^18, 20^ and podocyte-specific deletion of *β*-catenin protects from nephropathy.^19, 20^ Tankyrases have been shown to be efficient targets in inhibiting this signaling.^17^ Here, we show that CD2AP interacts with tankyrase 1 and tankyrase 2, and that in the absence of CD2AP tankyrase-mediated total PARylation in podocytes is increased. Podocytes lacking CD2AP accumulate active *β*-catenin consequently leading to upregulation of LEF1 and fibronectin, both associated with podocyte injury. Tankyrase inhibitor treatment reduces total PARylation and *β*-catenin activation in cultured podocytes, but, surprisingly, worsens kidney injury and increases mortality of zebrafish in the absence of CD2AP.

Increased PARylation in podocytes has been shown to have a role in glomerular injury associated with type 2 diabetes and general PARP inhibitors ameliorate this pathological situation.^29^ We found that tankyrases are phosporylated in podocytes and that the absence of CD2AP leads to increased total PARylation due to the activity of tankyrases. AutoPARylation of tankyrases was reduced in the absence of CD2AP, indicating that PARylation of tankyrase binding partners or unbound PAR would contribute to the overall increase in total PAR. In line with this, PARP1 PARylation was found modestly increased when CD2AP was depleted. The data also indicated that CD2AP is not PARylated. Very little is still known about the complex relationship between tankyrase phosphorylation, autoPARylation and PARylation activity and the data available on their relationship vary, apparently reflecting cell-type and interaction partner-dependent differences.^14, 15, 16, 23, 26, 27^ Most of the known interaction partners of tankyrases are acceptors of tankyrase PARylation.^14, 15, 23^ CD2AP bears similarity with few proteins, Fanconi anemia protein FANCD2 that controls genomic integrity,^34^ and GDP-mannose-4,6-dehydratase (GMD) that is required for fucose synthesis,^35^ which bind tankyrases and inhibit their activity, but are not PARylated.

**Figure 8:**
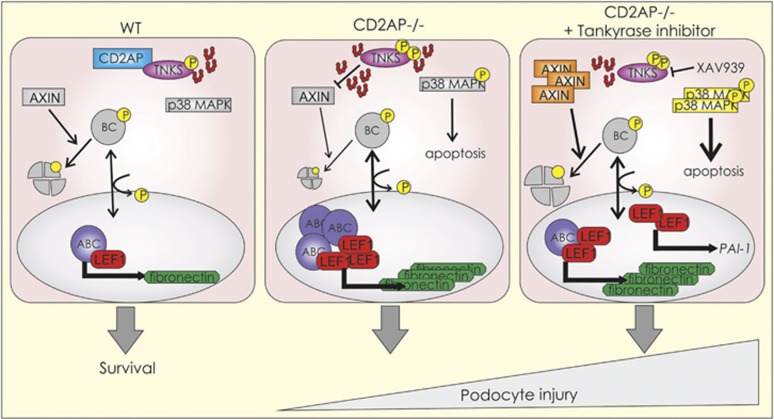
Schematic illustration of the molecular mechanisms contributing to injury of CD2AP-deficient podocytes upon tankyrase inhibition. In WT podocytes, CD2AP interacts with tankyrases (TNKS) and inhibits their activity. In CD2AP−/− podocytes, the activity of tankyrases is increased, leading to inhibition of beta-catenin (BC) degradation. Active beta-catenin (ABC) accumulates and translocates to the nucleus, where it together with LEF1 upregulates fibronectin. Tankyrase inhibition in CD2AP−/− podocytes leads to accumulation of AXIN1 and degradation of active beta-catenin. However, tankyrase inhibition fails to downregulate fibronectin, further upregulates LEF1 and enhances *PAI-1* expression. Additionally, phosphorylation of the pro-apoptotic p38 MAPK is enhanced. These events further aggravate injury in CD2AP-deficient podocytes

Our data indicate apparent dysregulation of the Wnt/*β*-catenin pathway in the absence of CD2AP. In cells deficient of CD2AP, active *β*-catenin is stabilized and translocated to the nucleus. This is coupled with upregulation of its downstream targets, LEF1 and fibronectin, whereas several other typical Wnt/*β*-catenin target genes associated with kidney injury were not affected. Notably, upregulation of LEF1 and fibronectin occurred without exogenic Wnt stimulation. Our data propose that the major enhancer of the pathway is enhanced tankyrase PARylation activity (Figure 8, CD2AP−/−). Interestingly, *MMP-9* was downregulated in the absence of CD2AP. Lack of CD2AP predisposes cells to apoptosis,^8^ and therefore reduced level of *MMP-9*, shown to protect tubular cells against apoptosis in acute kidney injury,^36^ could contribute to the susceptibility of CD2AP-deficient podocytes to apoptosis.

Inhibition of tankyrases has been shown to attenuate pathological conditions associated with activation of Wnt signaling, such as solid tumors^37, 38^ and fibrotic pulmonary diseases.^39, 40^ On the basis of this, targeting the Wnt/*β*-catenin pathway could also serve as a therapeutic strategy to treat a variety of proteinuric kidney diseases showing activation of the Wnt pathway, as also Wang *et al.*^18^ suggested. We found that treatment of CD2AP−/− podocytes with tankyrase inhibitor reduced the activity of *β*-catenin. However, it did not significantly reduce the increased expression of fibronectin, further increased the expression of LEF1, upregulated *PAI-1* (Figure 8, CD2AP−/−+tankyrase inhibitor), a fibrogenic factor that has been associated with the development of nephropathy,^41^ and increased pro-apoptotic signaling. Previously, it has been shown that upregulation of LEF1 coupled with accumulation of active *β*-catenin renders cancer cells unresponsive to tankyrase inhibition, which is due to LEF1 limiting the association of *β*-catenin with the destruction complex.^42^ Tankyrase inhibition in these situations leads as a forward regulatory loop to further upregulate LEF1. A situation similar to this could explain our findings in podocytes lacking CD2AP. Moreover, LEF1 and TCF-1 can be transcriptionally active also independently of *β*-catenin,^43^ and as *PAI-1* gene promoter harbors a functional TCF/LEF site,^22^ the observed PAI-1 upregulation can be a result of LEF1 activating the transcription of *PAI-1*. We thus propose a mechanism by which tankyrase inhibition aggravates podocyte injury in the absence of CD2AP: tankyrase inhibition leads to upregulation of LEF1, which in turn enhances transcription of *PAI-1* independently of *β*-catenin (Figure 8, CD2AP−/−+tankyrase inhibition). These events contribute to the detrimental consequences of tankyrase inhibition in the absence of CD2AP.

The harmful effects of inhibition of tankyrases in the absence of CD2AP were further supported in zebrafish larvae *in vivo*, in which knockdown of CD2AP coupled with inhibition of tankyrases increased the severity kidney injury and mortality. As both CD2AP and tankyrases are widely expressed,^2, 44^ may the phenotype reflect defects in the function of multiple tissues. The worsening of the kidney phenotype is a significant finding when considering treatment strategies for kidney diseases associated with impairment of CD2AP, such as FSGS.^45, 46^ Mutations in CD2AP observed in FSGS typically lead to reduced expression or absence of CD2AP or render CD2AP biologically defected.^45, 46^ This could lead to increased tankyrase activity in podocytes. Interestingly, the Wnt pathway has been shown to be activated in patients with primary or experimental FSGS,^47^ tempting to speculate that Wnt inhibition would be a beneficial treatment strategy for FSGS. Our findings, however, point out that inhibiting the Wnt pathway with tankyrase inhibitors in the absence of CD2AP could be deleterious.

In summary, our data show a novel mechanism that contributes to kidney injury and dysfunction in the absence of CD2AP. We show that total PARylation and the activity of *β*-catenin are elevated in CD2AP−/− podocytes, and that both can be inhibited by tankyrase-specific small molecule inhibitor. Tankyrase inhibition, however, further upregulates proteins known to associate with kidney injury, and aggravates kidney dysfunction and increases mortality in CD2AP-depleted zebrafish larvae. These findings highlight that inhibition of the Wnt/*β*-catenin pathway can have adverse effects and that tankyrase inhibitors are not a suitable approach to treat kidney diseases associated with lack of CD2AP.

## Materials and Methods

### Yeast two-hybrid screening

Construction of the rat glomerular yeast two-hybrid library and screening of the library using CD2AP as a bait have been described previously.^48, 49^

### Animals

Male Sprague-Dawley rats and male FVB mice were used to study tankyrases 1 and 2 expression in the kidney. Generation of CD2AP-knockout mouse has been described.^2^ Kidneys of the knockout mice and controls were collected at 3 weeks of age and used for analyses as described below. Zebrafish embryos were obtained from the breeding line of Turku strain and raised as described.^50^ Animal experiments were performed according to approved guidelines and were approved by the National Animal Experiment Board.

### Cell culture, transient transfections and inhibitor treatments

Conditionally immortalized podocytes derived from CD2AP−/− mice and WT littermates^8^ and HEK293T cells were maintained in DMEM containing 4.5 g/l glucose, 10% FCS, penicillin and streptomycin (Sigma-Aldrich, St. Louis, MO, USA). Podocyte cultures were supplemented with 10 U/ml IFN-*γ* (Sigma-Aldrich). HEK293T cells were transiently transfected with tankyrase 1 (kindly provided by Dr. Susan Smith, New York University School of Medicine, New York, NY, USA), flag-tagged tankyrase 2 and flag-tagged ankyrin domains of tankyrase 2 (kindly provided by Dr. Nai-Wen Chi, University of California, San Diego, La Jolla, CA, USA) using Lipofectamine 2000 (Invitrogen, Camarillo, CA, USA). Proliferating WT and CD2AP−/− podocytes were cultured to 70% confluency and treated with 3 *μ*M XAV939 (Sigma-Aldrich), 3 *μ*M ABT-888 (Selleckchem, Munich, Germany) or DMSO for 18 h when indicated.

### Antibodies

Antibodies used were rabbit anti-tankyrase 1/2 and mouse anti-Bcl-2 (Santa Cruz Biotechnology, Dallas, TX, USA), rabbit anti-podocin and mouse anti-tubulin (Sigma-Aldrich), goat anti-tankyrase 2, rabbit anti-BAX and rabbit anti-fibronectin (Abcam, Cambridge, UK), rabbit anti-CD2AP 1774 or 1764,^4, 51^ mouse anti-PARP1 and rabbit anti-Poly(ADP-ribose) (Enzo Life Sciences, Farmingdale, NY, USA), mouse anti-active-*β*-catenin (Millipore, Billerica, MA, USA), rabbit anti-LEF1 and rabbit anti-phospho p38 MAPK (Cell Signaling Technology, Danvers, MA, USA) and mouse anti-WT1 (Upstate, Lake Placid, NY, USA), guinea pig anti-p62/SQSTM1 (Progen Biotechnik GmbH, Heidelberg, Germany) and mouse anti-Caspase-1 (Adipogen, San Diego, CA, USA).

### Preparation of tissue and cell lysates and nuclear extracts

Glomerular and tubular fractions were isolated from rat kidney cortices using graded sieving.^52^ Tissues and cells were lysed as described.^53^ Pooled zebrafish larvae were sonicated in 50 mM Tris-HCl, pH 7.4, 150 mM NaCl, 1% Nonidet P-40, 0.5% sodium deoxycholate, 0.1% SDS supplemented with 1 mM PMSF and protease inhibitor cocktail (Roche, Basel, Switzerland). Nuclear extracts were prepared as described.^35, 54^

### Pull-down assay, immunoprecipitation and immunoblotting

Pull-down assay with glutathione-S-transferase (GST)-tagged SH3 domains^55^ or the NH_2_-terminus (amino acids 1–330) of mouse CD2AP was performed as described.^5^ Pull-down assay for PARylated proteins was performed with a WWE Affinity Resin kit (Tulip Biolabs, West Point, PA, USA) according to the manufacturer's instructions, and immunoprecipitation as described.^5^ Immunoblotting was performed as described.^51^

### Immunoperoxidase staining

Mouse kidneys were fixed with 10% formalin, dehydrated and embedded in paraffin. Immunoperoxidase staining was performed with a VectaStain Elite kit (Vector Laboratories, Burlingame, CA, USA) as described.^49^ Sections were analyzed with a Nikon Eclipse 800 microscope (Nikon, Tokyo, Japan).

### Immunofluorescence staining

Mouse kidney frozen sections (6 *μ*m) were fixed with acetone and cultured podocytes were fixed with 4% paraformaldehyde (PFA) and permeabilized with 0.1% Triton X-100. The stainings were carried out as described.^53^ AlexaFluor 594 donkey anti-rabbit and AlexaFluor 488 donkey anti-goat or donkey anti-mouse IgGs were used as secondary antibodies. Samples were analyzed with Leica TCS SP2 or SP8 confocal microscopes (Leica Microsystems, Wetzlar, Germany).

### PARylation assay

The total PARylation was measured with HT PARP *in vivo* Pharmacodynamic Assay II (Trevigen, Gaithersburg, MD, USA).

### Duolink *in situ* PLA

Duolink *in situ* PLA was carried out according to the manufacturer's instructions (Duolink, Olink Bioscience, Uppsala, Sweden). Fluorescence images were captured using Zeiss Axioplan2 microscope (Carl Zeill Microscopy, Thornwood, NY, USA). Quantification was performed using the Duolink Image Tool (Olink Bioscience).

### Quantitative RT-PCR

Quantitative PCR was performed as described in Wang *et al.*^56^ Primer sequences for *Snail1, MMP-9, FSP-1, PAI-1, LEF1, TCF-1, TCF-3* and *TCF-4* are shown in Supplementary Table S1. The expression levels of the studied molecules were normalized to *β*-actin using the comparative *C*_*t*_ method. Reverse transcriptions were performed with three individual RNA preparations and quantitative RT-PCRs were performed in triplicate.

### Knockdown and tankyrase inhibitor treatment in zebrafish

MO blocking translation of *cd2ap* (5′-CATACTCCACCACCACCTCAACCAT-3′)^32^ and a standard control MO were purchased from GeneTools, LLC (Philomath, OR, USA). Zebrafish embryos were injected with 4 nl of 100 *μ*M MOs using a Narishige MN-153 microinjector (Tokyo, Japan). Embryos were allowed to develop until 3 days post fertilization (dpf) and then transdermally exposed to 5 *μ*M XAV939 or an equivalent volume of DMSO (0.05%) in E3 medium for 2 days. The phenotype of larvae on 5 dpf was examined under a Leica dissecting microscope.

### Histology of zebrafish larvae

Zebrafish larvae at 5 dpf were fixed with 4% PFA in PBS overnight at 4 °C and embedded in JB-4 (Polysciences, Inc., Warrington, PA, USA). In all, 4 *μ*m sections were stained with hematoxylin and eosin.

### Statistical analysis

Statistical analysis was performed with one-way ANOVA. Results are presented as mean±S.E.M.

## Abbreviations

CD2AP: CD2-associated protein
CD2AP−/−: CD2AP knockout
FSP-1: fibroblast-specific protein 1
FSGS: focal segmental glomerulosclerosis
GST: glutathione-S-transferase
LEF1: lymphoid enhancer factor 1
MMP-9: matrix metalloproteinase 9
MO: morpholino antisense oligonucleotide
PAI-1: plasminogen activator inhibitor-1
PAR: poly(ADP-ribose)
PARP1: poly(ADP-ribose) polymerase 1
PARP2: poly(ADP-ribose) polymerase 2
PARylation: poly(ADP-ribosyl)ation
TCF: T-cell factor
TNKS1: tankyrase 1
TNKS2: tankyrase 2
WT: wild type
