# Supplementary material for: Tankyrase inhibition aggravates kidney injury in the absence of CD2AP
Source: Cell Death Dis. 2016 Jul 21;7(7):e2302–. doi: 10.1038/cddis.2016.217 (PMC4973355; doi:10.1038/cddis.2016.217)
Supplement: Supplementary Information [file cddis2016217x1.docx]

**Tankyrase inhibition aggravates kidney injury in the absence of CD2AP**

Sara Kuusela, Hong Wang, Anita A. Wasik, Hani Suleiman and Sanna Lehtonen

**SUPPLEMENTARY INFORMATION**

**Supplementary Figure S1. CD2AP-GST fusion proteins used for the pull-down assay and** **Western blot analysis of overexpression efficiency.** (a) GST-CD2AP fusion proteins covering the N-terminus or individual SH3-domains of CD2AP and GST alone were separated by 10% SDS-PAGE gel and stained with Page Blue protein staining solution (Thermo Scientific, Rockford, IL). The image shows the purity and integrity of the fusion proteins used for the pull-down assay. (b) HEK293T cells were transiently transfected with tankyrase 1, flag-tagged tankyrase 2 and flag- tagged ankyrin domains of tankyrase 2. Immunoblotting of cell lysates (30 µg) with antibodies against tankyrase 1/2 and flag confirms overexpression of the proteins. Tubulin was included as a loading control.

**Supplementary Figure S2. Tankyrase 1 is phosphorylated in WT and CD2AP-/- podocytes.** Wildtype (WT) and CD2AP-/- podocytes lysed in NP-40 buffer without phosphatase inhibitors were subjected to dephosphorylation treatment with calf intestinal alkaline phosphatase (CIP; New England BioLabs, Inc., Ipswich, MA) CIP treatment leads to an increase in the electrophoretic mobility of tankyrases as visualized by immunoblotting with an antibody recognizing both tankyrase 1 and tankyrase 2. Lysates were treated or not with CIP at 37˚C for 1 h.

**Supplementary Figure S3. ABT-888 inhibits hydrogen peroxide-induced PARylation in podocytes.** (**a, b, c**) Representative immunofluorescence images of podocytes treated with genotoxin hydrogen peroxide (H_2_O_2_; 20 mM, 20 min), fixed with acetone and stained with an antibody against PAR. (**a**) Untreated podocytes show weak staining for PAR. (**b**) H_2_O_2_-treatment induces PARylation in podocytes and (**c**) ABT-888-treatment (3 µM diluted in DMSO, 120 min) inhibits H_2_O_2_ –induced PARylation. DMSO was used as a diluent only -control (**a**). (**d**) Total PARylation assay (HT PARP *in vivo* Pharmacodynamic Assay II, Trevigen, Gaithersburg, MD) shows that H_2_O_2_-treatment induces PARylation in podocytes and ABT-888 treatment inhibits H_2_O_2_ –induced total PARylation. Bars show the mean and error bars the ±SEM of three independent experiments. P values were calculated with one-way ANOVA (***=P<0,001).

**Supplementary Figure S4. Total PARylation is increased in the kidneys of CD2AP-/- mice.** Immunofluorescent images of wildtype (WT) (**a, b, c)** and CD2AP-/- (**d, e, f**) kidney sections double labelled for PAR (**a, d**) and WT-1 (**b, e**) to label podocyte nuclei show an increase in PARylation in the absence of CD2AP. (**c, f**) show merged images of (**a, b**) and (**d, e**), respectively. Scale bar 50 µm.

**Supplementary Figure S5. Active β-catenin and LEF1 are upregulated in the absence of CD2AP.** Immunofluorescence images of wildtype (WT) (**a, b, c, d, e, f**) and CD2AP-/- (**g, h, i, j, k, l**) podocytes. Double labelling with active β-catenin (ABC) and LEF1 IgGs showed upregulation of active β-catenin and LEF1 in CD2AP-/- podocytes (**g, h, i**) compared to WT podocytes (**a, b, c**). (**d, e, f, j, k, l**) Immunofluorescence images of WT (**d, e, f**) and CD2AP-/- (**j, k, l**) podocytes treated with tankyrase inhibitor XAV939 reveals that XAV939-treatment downregulates active β-catenin in both WT and CD2AP-/- podocytes, but further upregulates LEF1 in CD2AP-/- podocytes when compared to non-treated podocytes. Scale bar: 25 µm.

**Supplementary Figure S6. Phenotype of *cd2ap* and control morpholino antisense oligonucleotide -treated larvae at 5 dpf, with and without tankyrase inhibitor XAV939 treatment.** Zebrafish embryos were injected with *cd2ap* (*cd2ap*-MO) or control (C-MO) morpholino antisense oligonucleotides, and then treated with tankyrase inhibitor XAV939 between 3 and 5 dpf. As a control, larvae were treated with DMSO.

**Supplementary Figure S7. XAV939 treatment reduces insulin-stimulated AKT phosphorylation in the absence of CD2AP.** Wildtype (WT) and CD2AP-/- podocytes with and without XAV939 treatment were starved and stimulated with 20 nM insulin for 20 min. (**a**) Immunoblotting of cell lysates (30 µg) with antibodies against phosphorylated AKT (pAKT, S473, rabbit anti-phosporylated AKT, Cell Signaling Technology, Danvers, MA) and total AKT (mouse anti-panAKT, R&D Systems, Minneapolis, MN) shows that WT and CD2AP-/- podocytes are responsive to insulin and phosphorylate AKT. (**b**) Quantification of phospho-AKT/pan-AKT ratio indicates reduction of insulin-stimulated AKT phosphorylation in CD2AP depleted podocytes treated with XAV939. Bars show the mean and error bars the ±SEM of three independent experiments. P values were calculated with one-way ANOVA (*=P<0,05).

**Supplementary Table 1 ⎮ qRT-PCR primers used in the study**

| **Gene** | **Forward, 5´- 3'** | **Reverse, 5´- 3'** |
| --- | --- | --- |
| *Snail1* | tgggccaacttcccaagcag | AGGTCGTGCAGACACAAGGC |
| *mmp9* | GTGCCGGAAGCGCTCATGTA | GCTGTCGGCTGTGGTTCAGT |
| *fsp1* | ACGGTTACCATGGCAAGACCC | TAGGCAGCTCCCTGGTCAGTA |
| *Pai1* | CCCATCAGGCCACCGACTTC | GGTTTTCCCCGCTGTGGTCA |
| *Lef1* | AACTCTGCGCCACCGATGAG | GTCTGACCACCTCATGCCCG |
| *Tcf1* | CAATCTGCTCATGCCCTACC | CTTGCTTCTGGCTGATGTCC |
| *Tcf3* | TGAAGGAAAGTGCAGCCATTA | TTTCCGGGCAAGCTCAT |
| *Tcf4* | TGGCTGGTCTGCACGGGATA | CAGGGGCCGCACCAGTTATT |
| *β-actin* | GATATCGCTGCGCTGGTCGT | TGGGCCTCGTCACCCACATA |
