# Supplementary figures and images for: Tankyrase inhibition aggravates kidney injury in the absence of CD2AP
Source: Cell Death Dis. 2016 Jul 21;7(7):e2302–. doi: 10.1038/cddis.2016.217 (PMC4973355; doi:10.1038/cddis.2016.217)

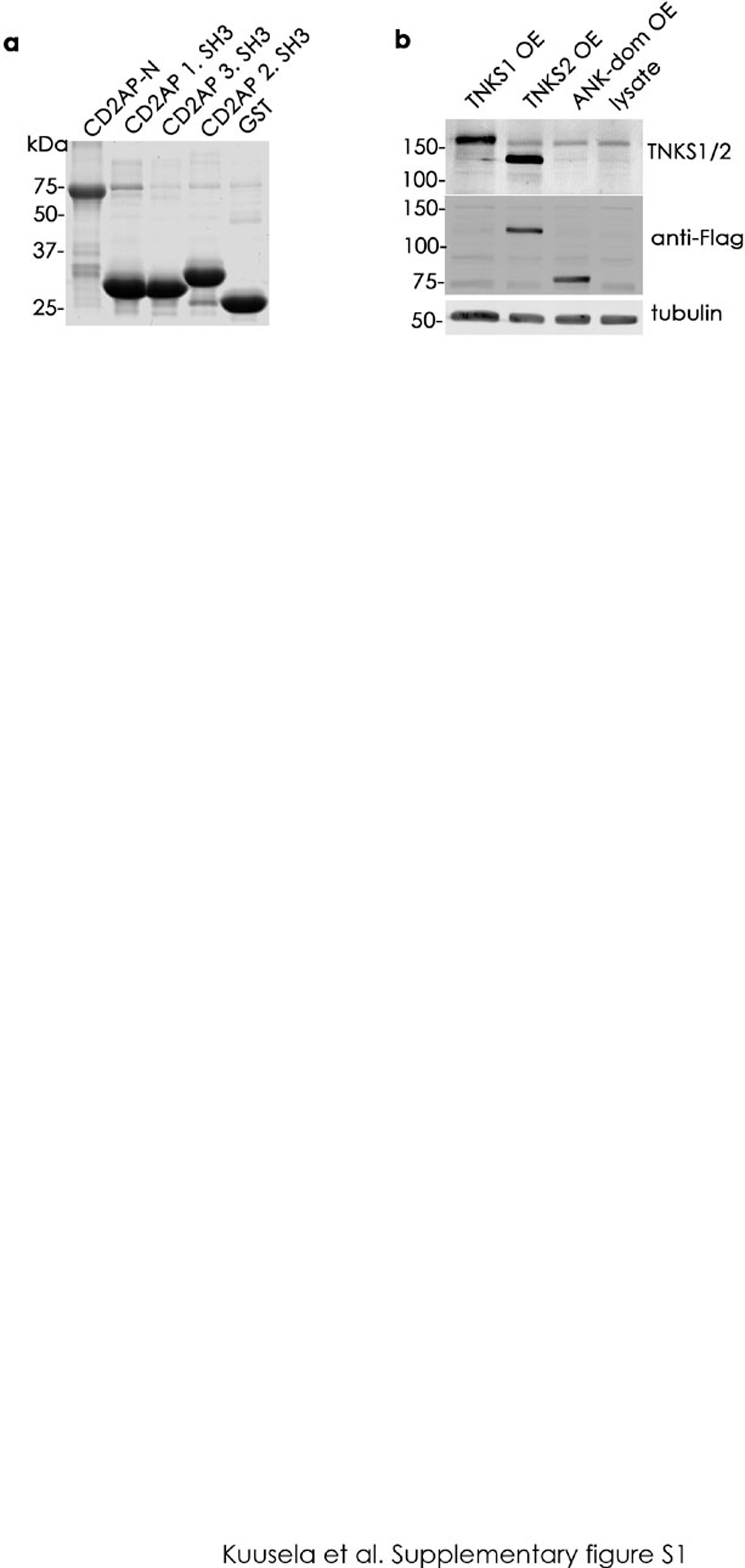

Supplement: Supplementary Figure S1 [file cddis2016217x2.tif]

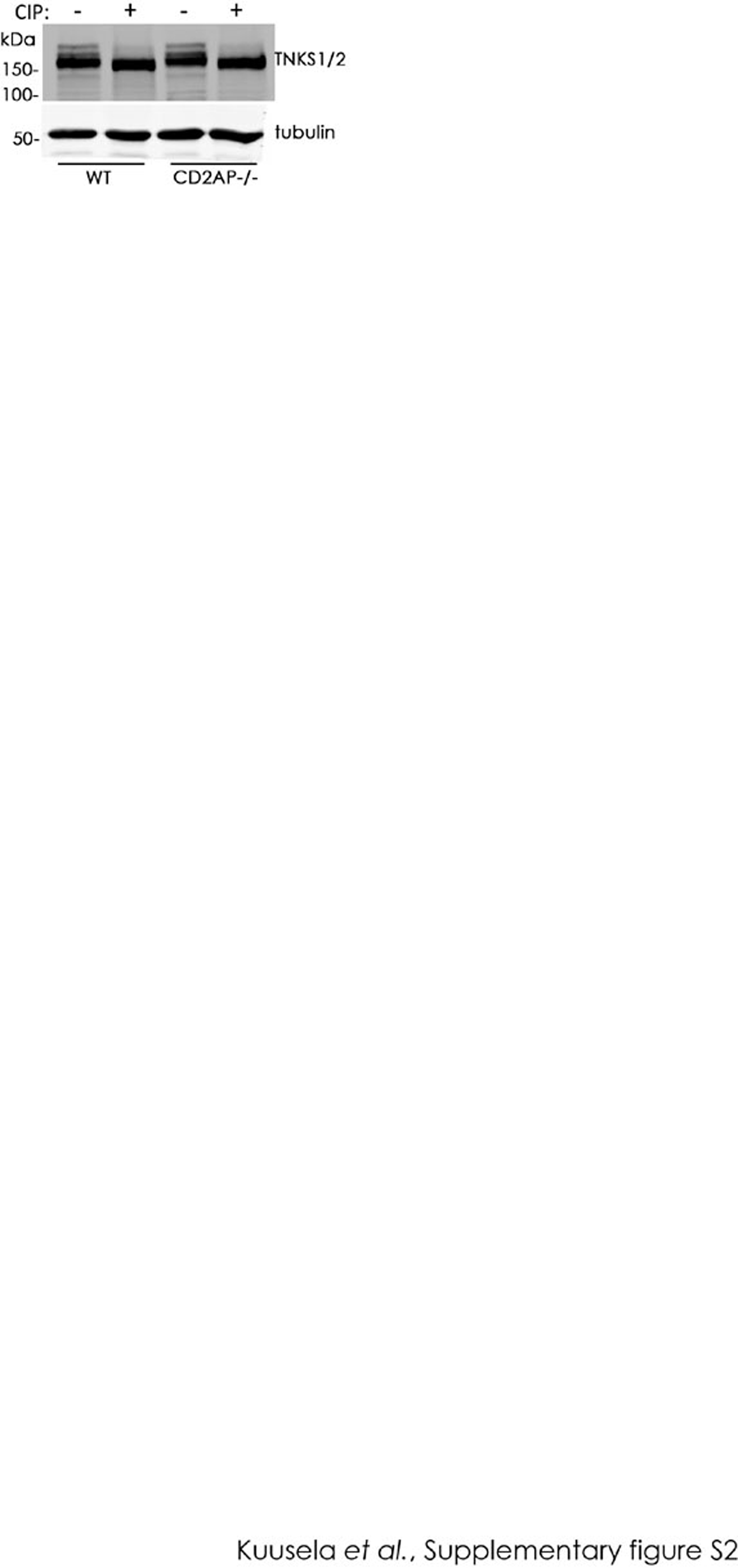

Supplement: Supplementary Figure S2 [file cddis2016217x3.tif]

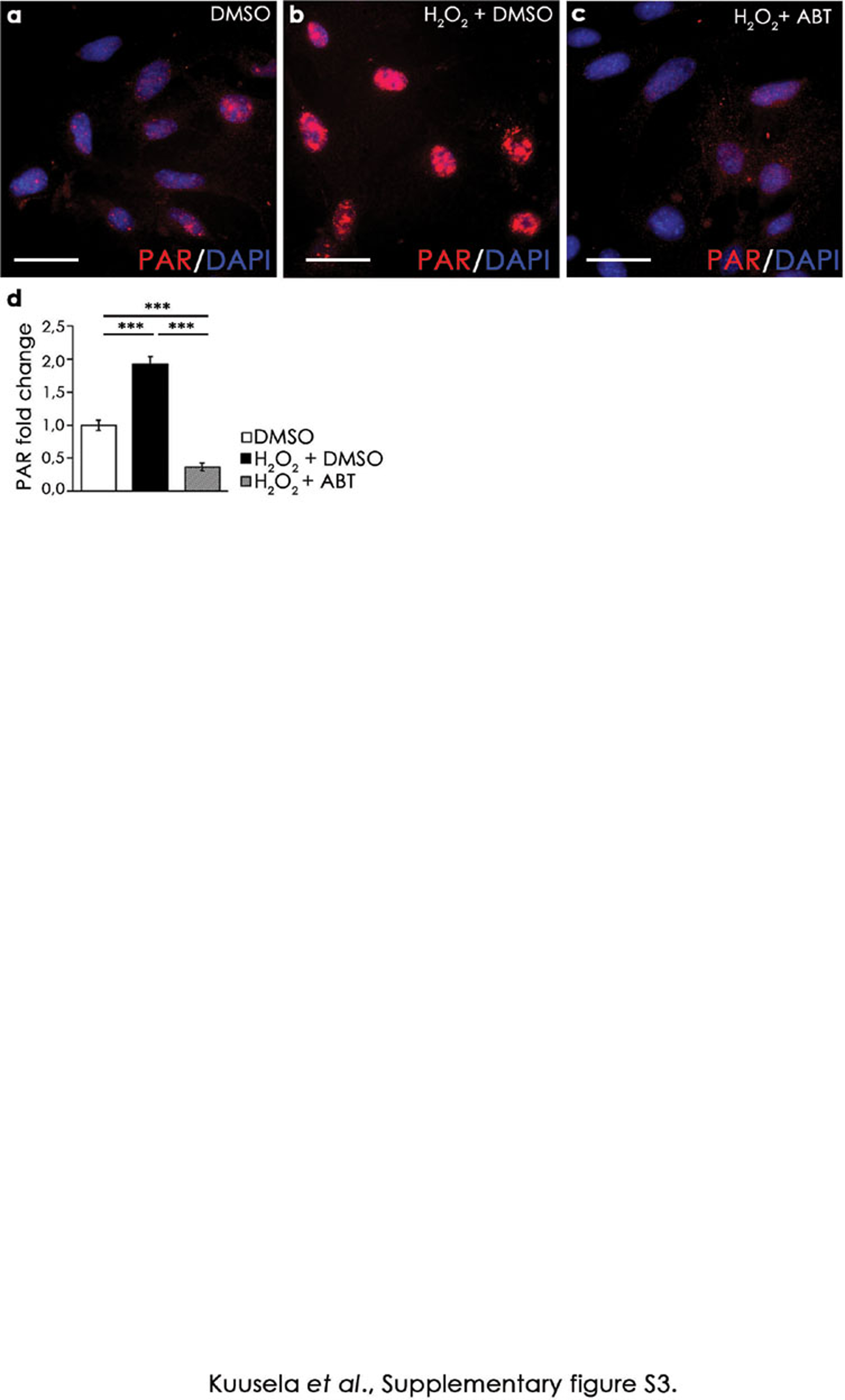

Supplement: Supplementary Figure S3 [file cddis2016217x4.tif]

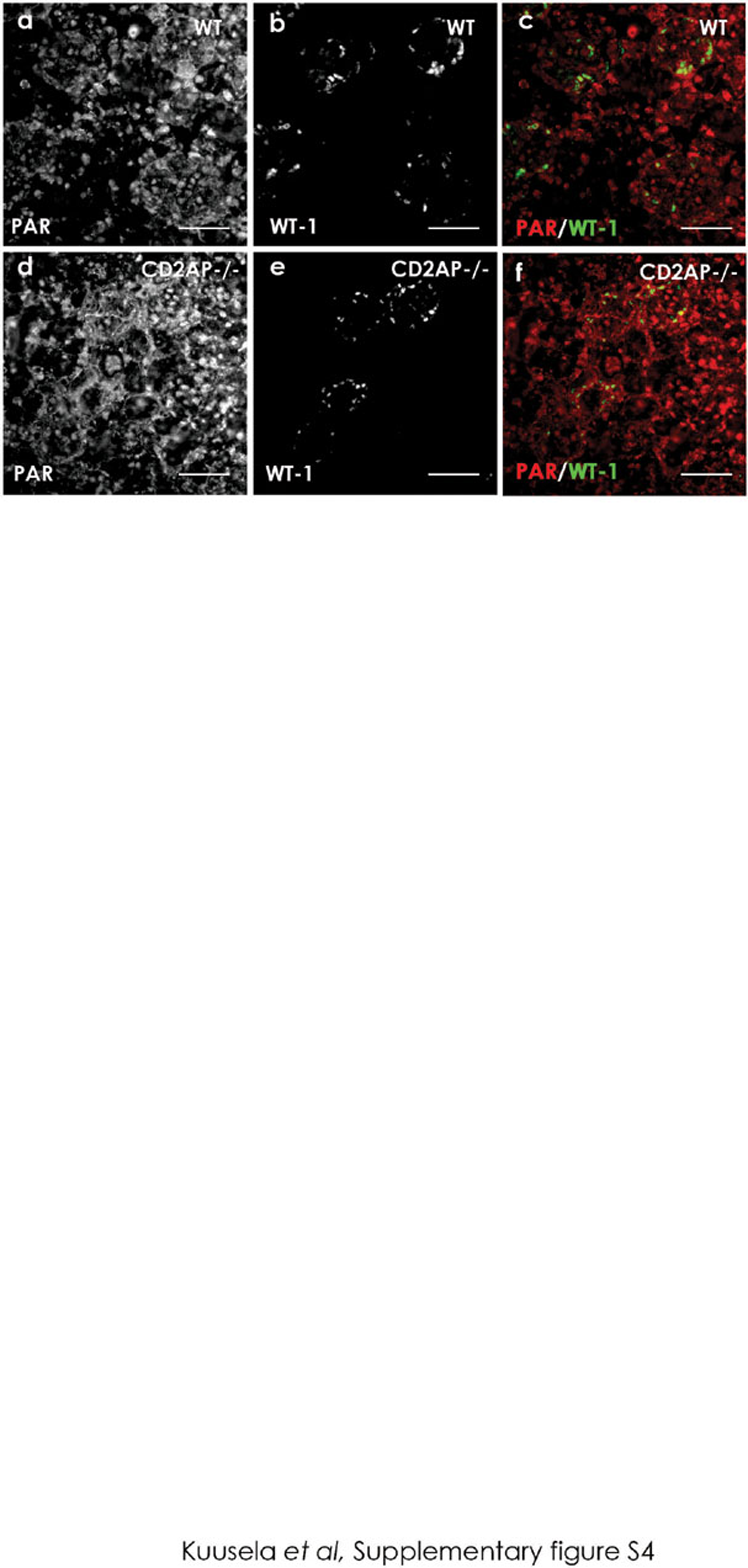

Supplement: Supplementary Figure S4 [file cddis2016217x5.tif]

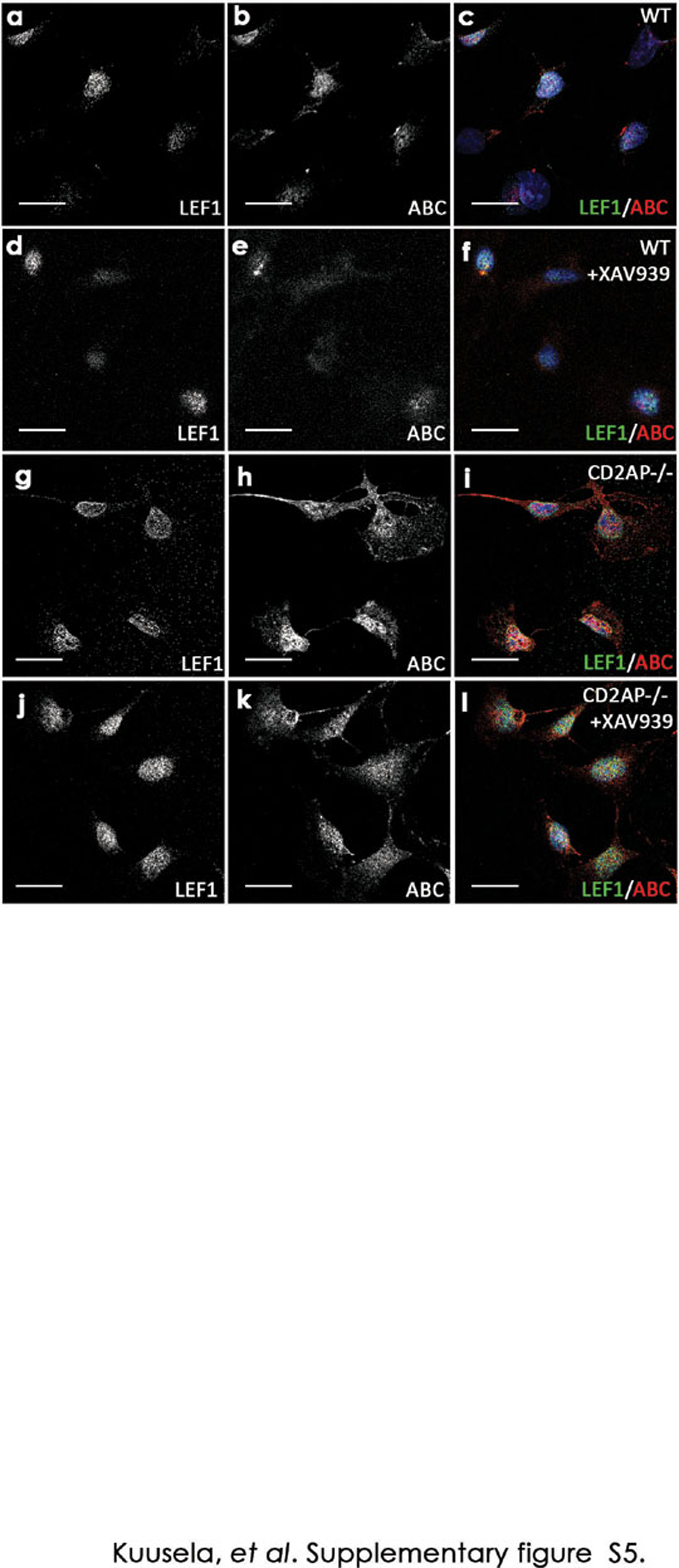

Supplement: Supplementary Figure S5 [file cddis2016217x6.tif]

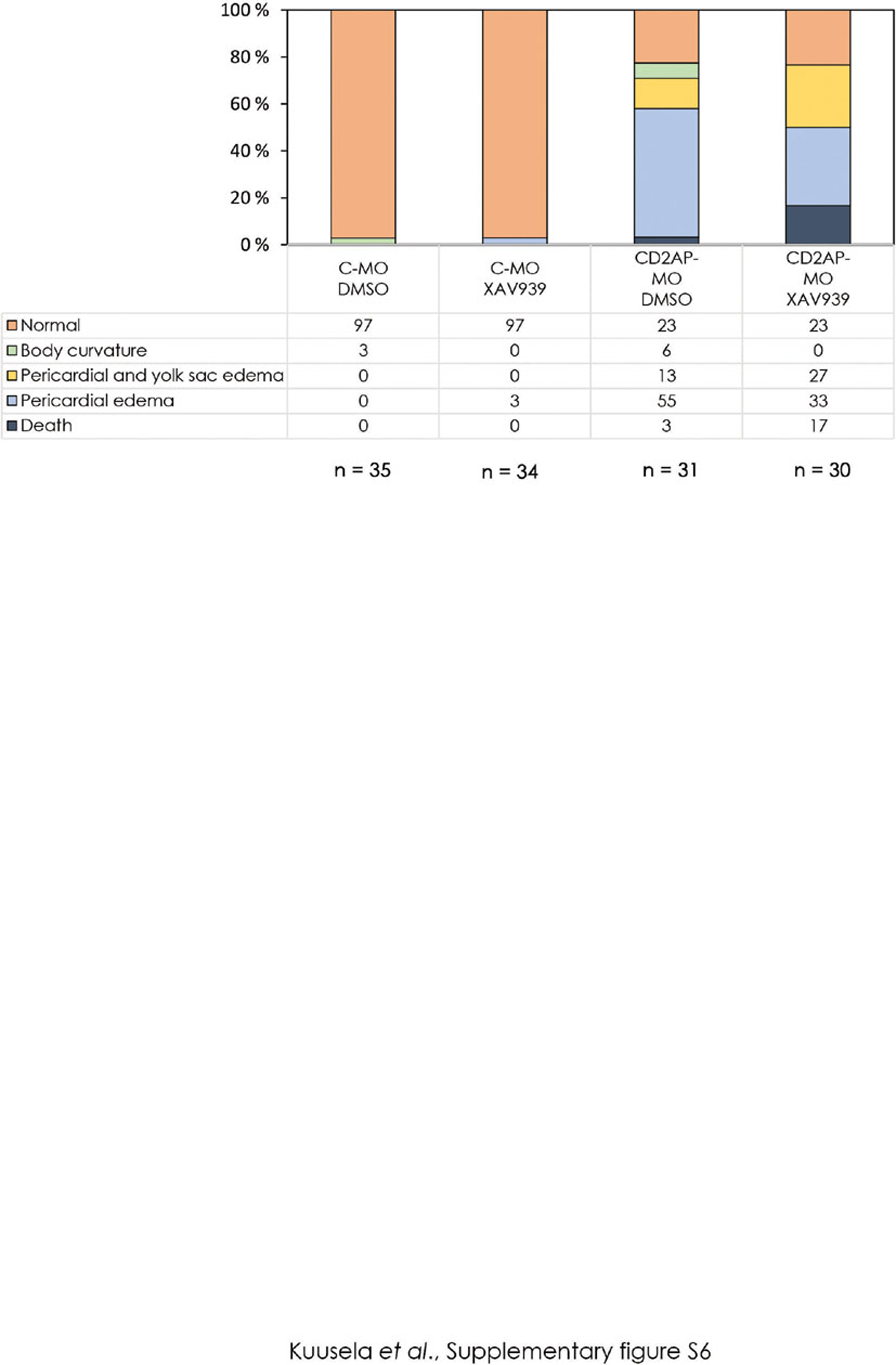

Supplement: Supplementary Figure S6 [file cddis2016217x7.tif]

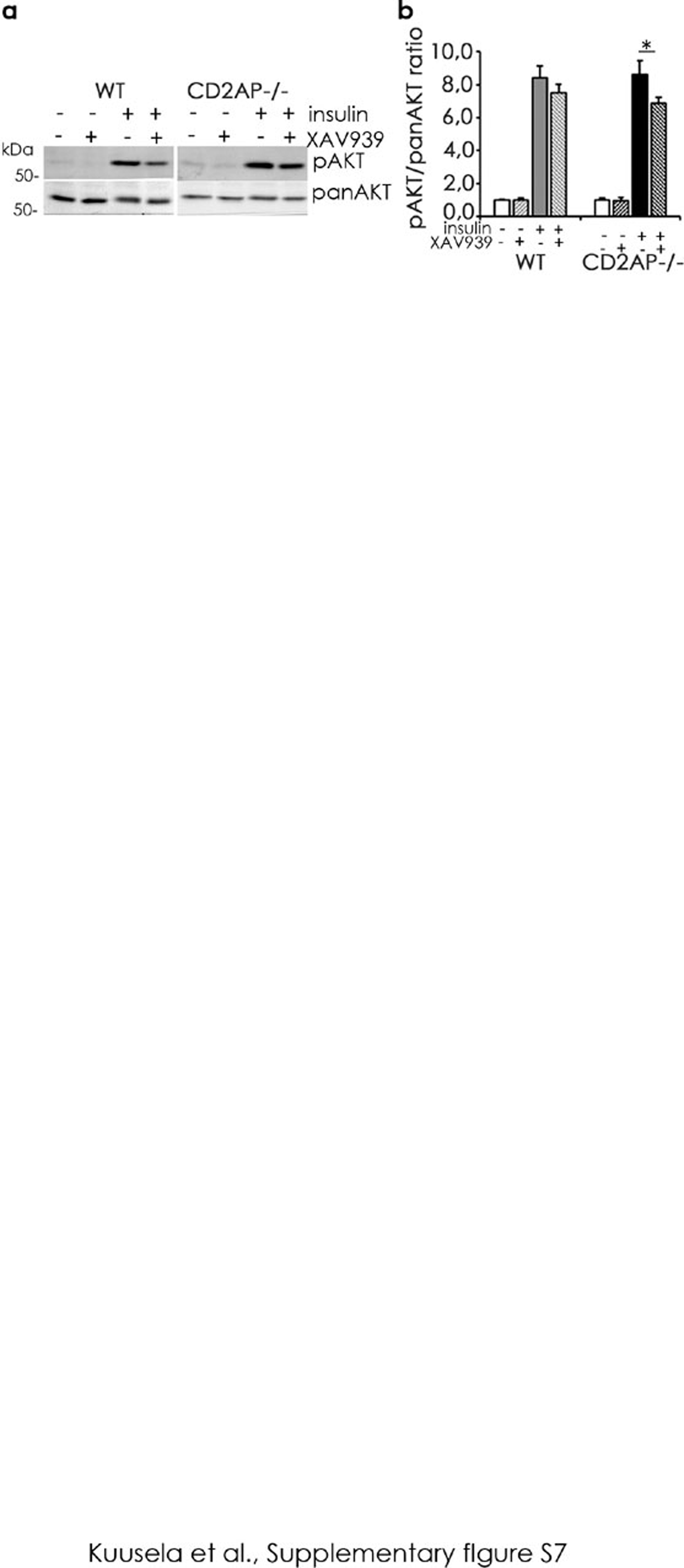

Supplement: Supplementary Figure S7 [file cddis2016217x8.tif]
